# Supplementary material for: Joint effect of alcohol drinking and tobacco smoking on all-cause mortality and premature death in China: A cohort study
Source: PLoS One. 2021 Jan 28;16(1):e0245670. doi: 10.1371/journal.pone.0245670 (PMC7842879; doi:10.1371/journal.pone.0245670)
Supplement: S1 Table — (DOCX) [file pone.0245670.s001.docx]

| **S1 Table Odds ratio of all-cause mortality among different smoking and drinking groups by sex** | | | | |
| --- | --- | --- | --- | --- |
|  | Nonsmoker/Nondrinker | Drinker | Smoker | Smoker/Drinker |
| **All participants** |  |  |  |  |
| Unadjusted | 1.00 | 0.99 (0.62, 1.56) | 1.75 (1.39, 2.19) | 1.28 (1.06, 1.54) |
| Adjusted* | 1.00 | 1.06 (0.61, 1.86) | 1.51 (1.09, 2.10) | 1.47 (1.03, 2.08) |
| **Male** |  |  |  |  |
| Unadjusted | 1.00 | 0.78 (0.37, 1.65) | 1.11 (0.77, 1.61) | 0.83 (0.60, 1.15) |
| Adjusted* | 1.00 | 1.18 (0.50, 2.80) | 1.46 (0.94, 2.26) | 1.42 (0.96, 2.10) |
| **Female** |  |  |  |  |
| Unadjusted | 1.00 | 0.89 (0.57, 1.37) | 2.34 (1.60, 3.43) | 3.14 (1.82, 5.41) |
| Adjusted* | 1.00 | 0.87 (0.50, 1.50) | 1.56 (0.99, 2.44) | 2.02 (0.95, 4.30) |
| *Adjusted variables include sex, age, middle school education, residence status, marital status, hypertension, dyslipidemia, diabetes, history of CVD, overweight or obesity. | | | | |
|  |  |  |  |  |
|  |  |  |  |  |
