# Supplementary material for: Joint effect of alcohol drinking and tobacco smoking on all-cause mortality and premature death in China: A cohort study
Source: PLoS One. 2021 Jan 28;16(1):e0245670. doi: 10.1371/journal.pone.0245670 (PMC7842879; doi:10.1371/journal.pone.0245670)
Supplement: S2 Table — (DOCX) [file pone.0245670.s002.docx]

| **S2 Table Odds ratio of premature death among different smoking and drinking groups by sex^1^** | | | | |
| --- | --- | --- | --- | --- |
|  | **Nonsmoker/Nondrinker** | **Drinker** | **Smoker** | **Smoker/Drinker** |
| **All participants** |  |  |  |  |
| Unadjusted | 1.00 | 0.84 (0.48, 1.46) | 1.49 (0.90, 2.45) | 2.12 (1.46, 3.08) |
| Adjusted ^2^ | 1.00 | 1.11 (0.57, 2.15) | 2.17 (1.11, 4.22) | 3.14 (1.56, 6.34) |
| **Male** |  |  |  |  |
| Unadjusted | 1.00 | 0.29 (0.11, 0.83) | 0.69 (0.28, 1.68) | 1.01 (0.46, 2.22) |
| Adjusted ^2^ | 1.00 | 0.50 (0.17, 1.47) | 1.17 (0.46, 2.93) | 1.57 (0.71, 3.47) |
| **Female** |  |  |  |  |
| Unadjusted | 1.00 | 1.31 (0.66, 2.61) | 2.20 (0.99, 4.89) | 5.36 (2.32, 12.36) |
| Adjusted ^2^ | 1.00 | 1.25 (0.59, 2.66) | 2.01 (0.83, 4.83) | 4.95 (2.00, 12.27) |

^1^ Premature death was defined as mortality before age 72.7 years in men and 76.9 years in women, which were the average life expectancies in China in 2011; however, age of death can only be estimated among CHALS 2013 follow-up.

^2^ Adjusted variables include sex, age, middle school education, residence status, marital status, hypertension, dyslipidemia, diabetes, history of CVD, overweight or obesity.
