# Supplementary material for: Joint effect of alcohol drinking and tobacco smoking on all-cause mortality and premature death in China: A cohort study
Source: PLoS One. 2021 Jan 28;16(1):e0245670. doi: 10.1371/journal.pone.0245670 (PMC7842879; doi:10.1371/journal.pone.0245670)
Supplement: S3 Table — (DOCX) [file pone.0245670.s003.docx]

**S3 Table Comparison of baseline characteristics between participants with and without missing data**

| **Variable** | **Excluded (n=171)** | **Included (n=17080)** | ***P*** |
| --- | --- | --- | --- |
| Age, mean (SD) | 61.16 (12.28) | 59.58 (9.86) | 0.12 |
| Missing | 26 | 0 |  |
| Gender, n (%) |  |  | 0.33 |
| Male | 70 (44.9) | 8332 (48.8) |  |
| Female | 86 (55.1) | 8748 (51.2) |  |
| Missing | 15 | 0 |  |
| Highest level of education, n (%) |  |  | 0.38 |
| Illiterate | 32 (22.9) | 4696 (27.5) |  |
| Primary School | 55 (39.3) | 6678 (39.2) |  |
| Middle School | 47 (33.6) | 5268 (30.9) |  |
| Tertiary education | 6 (4.3) | 414 (2.4) |  |
| Missing | 28 | 24 |  |
| Current residence status, n (%) |  |  | 0.89 |
| No | 48 (34.0) | 3733 (21.9) |  |
| Yes | 93 (66.0) | 13334 (78.1) |  |
| Missing | 30 | 13 |  |
| Marital status, n (%) |  |  | 0.003 |
| Married | 108 (77.7) | 14852 (87.0) |  |
| Not married | 31 (22.3) | 2227 (13.0) |  |
| Missing | 32 | 1 |  |
| Hypertension, n (%) |  |  | <0.001 |
| Yes | 19 (11.3) | 6567 (38.4) |  |
| No | 149 (88.7) | 10513 (61.6) |  |
| Missing | 3 | 0 |  |
| Dyslipidemia, n (%) |  |  | <0.001 |
| Yes | 28 (16.7) | 5418 (31.7) |  |
| No | 140 (83.3) | 11662 (68.3) |  |
| Missing | 3 | 0 |  |
| Diabetes, n (%) |  |  | 0.15 |
| Yes | 8 (4.8) | 1286 (7.5) |  |
| No | 160 (95.2) | 15794 (92.5) |  |
| Missing | 3 | 0 |  |
| CVD, n (%) |  |  | <0.001 |
| Yes | 8 (4.8) | 2371 (13.9) |  |
| No | 160 (95.2) | 14709 (86.1) |  |
| Missing | 3 | 0 |  |
| Overweight or obesity, n (%) |  |  | 0.34 |
| No | 16 (69.6) | 7927 (59.9) |  |
| Yes | 7 (30.4) | 5304 (40.1) |  |
| Missing | 148 | 3849 |  |
| Smoking, n (%) |  |  | 0.83 |
| No | 13 (61.9) | 10188 (59.6) |  |
| Yes | 8 (38.1) | 6892 (40.4) |  |
| Missing | 150 | 0 |  |
| Drink status, n (%) |  |  | 0.30 |
| No | 4 (26.7) | 6742 (39.5) |  |
| Yes | 11 (73.3) | 10338 (60.5) |  |
| Missing | 156 | 0 |  |
