# Supplementary material for: Joint effect of alcohol drinking and tobacco smoking on all-cause mortality and premature death in China: A cohort study
Source: PLoS One. 2021 Jan 28;16(1):e0245670. doi: 10.1371/journal.pone.0245670 (PMC7842879; doi:10.1371/journal.pone.0245670)
Supplement: S4 Table — (DOCX) [file pone.0245670.s004.docx]

**S4 Table Name of variables and datasets of CHARLS data used in this paper**

| **Variables** | **Name of datasets** | **Year of survey** |
| --- | --- | --- |
| id | demographic_background | 2011, baseline |
| ind_weight_ad2 | weight | 2011, baseline |
| da010_2_s1 | health_status_and_functioning | 2011, baseline |
| da010_2_s2 | health_status_and_functioning | 2011, baseline |
| da010_2_s3 | health_status_and_functioning | 2011, baseline |
| da059 | health_status_and_functioning | 2011, baseline |
| da060 | health_status_and_functioning | 2011, baseline |
| da061 | health_status_and_functioning | 2011, baseline |
| da062_1 | health_status_and_functioning | 2011, baseline |
| da062_2 | health_status_and_functioning | 2011, baseline |
| da062s1 | health_status_and_functioning | 2011, baseline |
| da062s2 | health_status_and_functioning | 2011, baseline |
| da063 | health_status_and_functioning | 2011, baseline |
| da064 | health_status_and_functioning | 2011, baseline |
| da065_1 | health_status_and_functioning | 2011, baseline |
| da065_2 | health_status_and_functioning | 2011, baseline |
| da065s1 | health_status_and_functioning | 2011, baseline |
| da065s2 | health_status_and_functioning | 2011, baseline |
| da066 | health_status_and_functioning | 2011, baseline |
| da067 | health_status_and_functioning | 2011, baseline |
| DA069 | health_status_and_functioning | 2011, baseline |
| exb001_2_2013 | Exit_Interview | 2013, follow-up |
| exb001_1_2013 | Exit_Interview | 2013, follow-up |
| be001 | demographic_background | 2011, baseline |
| bc001 | demographic_background | 2011, baseline |
| ba004 | demographic_background | 2011, baseline |
| ba002_1 | demographic_background | 2011, baseline |
| ba002_2 | demographic_background | 2011, baseline |
| ba002_3 | demographic_background | 2011, baseline |
| rgender | demographic_background | 2011, baseline |
| bd001 | demographic_background | 2011, baseline |
| da007_1_ | health_status_and_functioning | 2011, baseline |
| da007_2_ | health_status_and_functioning | 2011, baseline |
| da007_3_ | health_status_and_functioning | 2011, baseline |
| da007_4_ | health_status_and_functioning | 2011, baseline |
| da007_5_ | health_status_and_functioning | 2011, baseline |
| da007_6_ | health_status_and_functioning | 2011, baseline |
| da007_7_ | health_status_and_functioning | 2011, baseline |
| da007_8_ | health_status_and_functioning | 2011, baseline |
| da007_9_ | health_status_and_functioning | 2011, baseline |
| da007_10_ | health_status_and_functioning | 2011, baseline |
| da007_11_ | health_status_and_functioning | 2011, baseline |
| da007_12_ | health_status_and_functioning | 2011, baseline |
| da007_13_ | health_status_and_functioning | 2011, baseline |
| da007_14_ | health_status_and_functioning | 2011, baseline |
| ql002 | biomarkers | 2011, baseline |
| qi002 | biomarkers | 2011, baseline |
| newhba1c | blood | 2011, baseline |
| da014s1 | health_status_and_functioning | 2011, baseline |
| da014s2 | health_status_and_functioning | 2011, baseline |
| da014s3 | health_status_and_functioning | 2011, baseline |
| da007_1_ | health_status_and_functioning | 2011, baseline |
| da011s1 | health_status_and_functioning | 2011, baseline |
| da011s2 | health_status_and_functioning | 2011, baseline |
| qa003 | biomarkers | 2011, baseline |
| qa007 | biomarkers | 2011, baseline |
| qa011 | biomarkers | 2011, baseline |
| qa004 | biomarkers | 2011, baseline |
| qa008 | biomarkers | 2011, baseline |
| qa0012 | biomarkers | 2011, baseline |
| newcho | blood | 2011, baseline |
| newldl | blood | 2011, baseline |
| newtg | blood | 2011, baseline |
| newhdl | blood | 2011, baseline |
| da007_2_ | health_status_and_functioning | 2011, baseline |
| da010_2_s1 | health_status_and_functioning | 2011, baseline |
| da010_2_s2 | health_status_and_functioning | 2011, baseline |
| da010_2_s3 | health_status_and_functioning | 2011, baseline |
