# Supplementary material for: Joint effect of alcohol drinking and tobacco smoking on all-cause mortality and premature death in China: A cohort study
Source: PLoS One. 2021 Jan 28;16(1):e0245670. doi: 10.1371/journal.pone.0245670 (PMC7842879; doi:10.1371/journal.pone.0245670)
Supplement: Attachment — Submitted filename: Response to Reviewers.docx [file pone.0245670.s007.docx]

Review Comments to the Author

Reviewer #1:

**Q1:** This is an interesting paper on the combined effect of alcohol consumption and tobacco use on mortality and premature death in China. However, I see some major limitations to the paper. I see in your discussion section you have mentioned that the paper is limited by not having information on the amount that participants drunk. This is a major limitation. And the definition of at least once a month seems small. Having moderate, heavy drinking would really add to the paper. Is there some other way of modelling this from other studies/datasets? Also not having information on cause of mortality limits the results of this paper significantly.

**Answer:** *Thanks, we totally agreed with your comments that lack of information on the amount of drinking do limit our findings. However, the CHARLS data are the only data we got. In this database, the definition of at least once a month of drinking was the only and the largest of frequencies of drinking option.*

*Related question: Did you ever drink alcoholic beverages in the past? How often?*

*1. I never had a drink;*

*2. I used to drink less than once a month;*

*3.* *I used to drink more than once a month.*

*We have add this as a limitation of our study. (Page 17 Line 207-211)*

**Q2:** Are you able to look at different levels of tobacco use and drinking? Currently the paper only looks at "ever" smoking, 100 cigarettes in their lifetime. It would useful also to look smoking frequency. Is this possible?

**Answer:** *Thank you for the constructive suggestion. The following table is the results of smoking quantity (pack years) and mortality. Although this result looks good, we still prefer not to report in this paper for the following reasons: 1. small number of events (death) been followed because of the short follow-up duration limited reliability to estimate the association between smoking quantity in subgroups and mortality. 2. Aim of this paper was to estimate the joint effect of smoking and drinking on death, the following table is suitable for the topic of the effect of smoking quality on mortality, therefore, we are preparing for another paper according to the results from the following table. Finally, really thanks again for your constructive suggestion.*

Table Odds ratio of total or premature death among different smoking groups, as compared with non-smokers in total and subgroup analysis ^1^

| **Smoking quantity (pack years)** | **Overall** | **Men** | **Women** |
| --- | --- | --- | --- |
| Total death |  |  |  |
| 0 | 1.00 | 1.00 | 1.00 |
| <15 | 0.77 (0.43, 1.40) | 0.68 (0.36, 1.29) | 1.06 (0.43, 2.62) |
| 15-30 | 1.27 (0.73, 2.22) | 1.24 (0.72, 2.13) | 1.09 (0.36, 3.32) |
| ≥30 | 1.45 (0.84, 2.52) | 1.36 (0.82, 2.25) | 2.53 (0.99, 6.44) |
| Type 3 test | 0.06 | 0.05 | 0.28 |
| Premature death |  |  |  |
| 0 | 1.00 | 1.00 | 1.00 |
| <15 | 1.46 (0.73, 2.90) | 1.60 (0.73, 3.52) | 0.55 (0.07, 4.27) |
| 15-30 | 2.35 (1.11, 4.94) | 2.29 (1.00, 5.25) | 1.81 (0.27, 12.43) |
| ≥30 | 2.16 (1.33, 4.13) | 2.06 (1.01, 4.22) | 2.85 (0.70, 11.60) |
| Type 3 test | 0.07 | 0.17 | 0.41 |

^1^ Adjusted variables include sex, age, middle school education, residence status, marital status, hypertension, dyslipidemia, diabetes, history of CVD, drinking status, overweight or obesity.

**Q3:** The manuscript requires a major edit for grammar. I would note some down but without line numbers it is very difficult to do this.

**Answer:** *Thank you for pointing out the language problems, we have revised the language thought out the paper and paid for the language editing service to ensure this paper meets the requirement of publication.*

**Q4:** The term tobacco smoke, isn't quite right. Tobacco smoking is more suitable in most places. The background sentence in the abstract doesn't make sense. Something like "Tobacco smoking and alcohol use have been shown to be associated with several diseases, however few studies have looked at the combined effect of smoking and drinking" may be more suitable.

**Answer:** *Yes, we changed “tobacco smoke” to “tobacco smoking” throughout the paper. (eg, Page 2 Line 24, Page 3 Line 50,…… Page 16 Line 171…...)*

**Q5:** Abstract in the results you don't need a "the" before confounders.

**Answer:** *Yes, we deleted the “the” in the Abstract. (Page 2, Line 33.)*

**Q6:** I would use "sex" rather than "gender" in the manuscript and keep this consistent throughout.

**Answer:** *Thank you for kindly suggestion, we changed “sex” to “gender” throughout the paper. (eg, Page 4 Line 82, Page 5 Line 113…... Page 17 Line 200……)*

**Q7:** I would find it easier to call the groups: non-smoker/non-drinker, smoker, drinker, smoker/drinker. Define it earlier on, but it is confusing to read as it is.

**Answer:** *Yes, we revised the name of the groups throughout the paper including the tables according to your suggestion. (eg, Page 5 Line 99-100……, Table 1-3.)*

**Q8:** Discussion, third line down you say non-smoker twice.

**Answer:** *Yes, we deleted the “non-smoker” accordingly. (eg, Page 16 Line 171-172.)*

Reviewer #2:

**Q1:** Be more careful with phrasings that insinuate causality. Present study is representative cohort design. Due to nature of observational study, the results did not showed causality Causal language (including use of terms such as "effect," "efficacy," "cause," and "x increased y") should be used only for randomized trials. For all other study designs (including meta-analyses of randomized trials), methods and results should be described in terms of association or correlation (eg, "x associated with an increase in y") and should avoid cause-and-effect wording.

**Answer:** *Thank you for reminding us of the inappropriate phrasings. We changed “there was a 2.4-fold (Unadjusted analysis: OR=2.34; 95%CI: 1.60-3.43) increase in the odds of all-cause mortality for smoker in female” to “**smoker in female was associated with a 2.4-fold* *(Unadjusted analysis:* *OR=2.34; 95%CI: 1.60-3.43) odds of all-cause mortality”, and other similar sentences were revised throughout the paper (eg, Page 2 Line 35-38, Page 12 Line 143-145, Line 152-155…...).*

**Q2:** Recent, the 4th wave of CHARLS (2018) has been released. The data can be updated using 4 waves from 2011 to 2018.

**Answer:** *Thanks, we noticed the 4^th^ wave of CHARLS had been released. But in the 4^th^ wave of data, the death information was not released. Therefore, the results in this paper were not influenced. Recently, some other research work is undergoing by using the 4^th^ wave of CHARLS data. Thank you so much for reminding.*

**Q3:** This study have many language/grammar errors. For example, in the section of results of abstract, ’smoker and drinker increased the odds of premature death’.

**Answer:** *Thanks, we firstly revised the language problems throughout the paper and then paid for the language editing service to ensure this paper meets the requirement of publication. The language error you mentioned in the abstract was also revised to “…the OR of smokers/drinkers for premature death was…” accordingly (Page 2, Line 35-36.)*

**Q4:** Please add the definition of outcome in the section of methods. (Premature death defined as mortality before age 72.7 years in men and 76.9 years in women, which were the average life expectancies in China in 2011).

**Answer:** *Thank you for the comments, we have added the definition of premature death in the methods section. (Page 5 Line 106-108.)*

**Q5:** The authors stated that they used weighted logistic regression. I am looking forward to the code for validating their correctness of analysis.

**Answer:** *Thank you for kindly help, the following codes were used in this paper (variable ind_weight_ad2 means individual weight with household and individual non-response adjustment). We hope our analysis is convincing.*

```
proc surveylogistic data=death;
	strata county village/list;
	class smok_drin(ref='0')/param=ref;
	model death=smok_drin;
	weight ind_weight_ad2;
run;
```

**Q6:** More sensitivity analysis need to be conducted to show the robustness of results. For example, excluding individuals who had events in the first five years of follow-up to avoid possible inverse causal relationships and excluding individuals who had events in the first ten years of follow-up to avoid possible inverse causal relationships.

**Answer:** *Thank you for the constructive suggestion. In this paper, we only get access to the CHARLS dataset recently, that means only the data released in the year of 2013 contained the death information, therefore, the sensitivity analysis after excluding baseline participants who had events in the first five years of follow-up was not applicable because of the relatively* *short follow-up duration (baseline survey at 2011 and death information been followed at 2013). In addition, we planned to conduct subgroup analysis to ensure the reliability of results, but limited by the small samples of death events. We have added related statement in the limitation paragraph. (Page 18, Line 215-220.)*

**Q7:** Please add the proportion of excluded missing data. There should be a mentioning, whether missing of data occurred at random.

**Answer:** *Thank you for the suggestion, we have added the related results (S1 Table).*

**Q8:** Please add the description of lost to follow-up.

**Answer:** *Thank you for the helpful suggestion, we have described the follow-up rate of CHARLS in 2013 accordingly. (Page 5, Line 102-105.)*
